# MET/HGF Signaling Pathway in Ovarian Carcinoma: Clinical Implications and Future Direction

**DOI:** 10.1155/2012/960327

**Published:** 2012-12-25

**Authors:** Paulette Mhawech-Fauceglia, Michelle Afkhami, Tanja Pejovic

**Affiliations:** ^1^Department of Pathology, University of Southern California, Room 7A116, 1200 N. State Street, Los Angeles, CA 90033-5000, USA; ^2^Department of Gynecologic Oncology and the Knight Cancer Institute, Oregon Health & Science University, Portland, OR 97239-3098, USA

## Abstract

The HGF/MET signaling pathway is abnormal in numerous cancers including ovarian cancer. MET is expressed in 70% of human cancer and it is overexpressed in 30% of ovarian cases and cancer cell lines. The HGF/MET pathway plays a role in the initiation and progression of ovarian cancer through the most distinctive biologic program known as “invasive growth” which is accomplished through a coordinated activation of cell motility, invasiveness, degradation of extracellular matrix, survival, and proliferation. Because of its ubiquitous role in cancer, the MET axis seems to be an attractive target for cancer therapy. Numerous HGF/MET pathway inhibitor compounds are already in use in clinical trials in various solid tumors. In this paper, we will discuss the HGF/MET pathway in ovarian cancer, its clinical significance, and its potential use as a target therapy in the future.

## 1. Introduction

Epithelial ovarian cancer (EOC) is the leading cause of death due to gynecologic malignancies in women in the United States, with 21,990 new cases and 15,460 women estimated to die of ovarian cancer in 2011 [1]. Epithelial ovarian cancers are divided into numerous histologic subtypes: serous, endometrioid, clear cell, mucinous, transitional cell, and squamous cell carcinomas with serous being the most common subtype representing 70–80% of all cases. Patients with ovarian cancer have high mortality rate due to the fact that the majority (almost 75%) present at an advanced stage disease with wide peritoneal metastasis. This mode of disease spread is explained by the fact that ovarian cancer mainly spreads by direct extension, through seeding or exfoliation of tumor cells from ovarian/fallopian tubes to the peritoneal cavity and it less likely to disseminate through vasculature, even though lymph nodes can be involved, which makes ovarian cancer a very aggressive disease [2]. The standard treatment for advanced stage disease is staging laparotomy with tumor debulking followed by platinum-taxane based chemotherapy [3]. Approximately 70–80% of patients with ovarian cancer will relapse after first-line chemotherapy and the management of relapsed ovarian carcinoma remains a difficult problem open to research [4]. Most patients will eventually die of chemotherapy resistant disease [5–7]. The overall survival rates remain disappointing with a little improvement in response rates, disease-free interval- and median survival rates. Therefore, novel therapies targeting DNA repair genes, tyrosine kinase inhibitors, angiogenesis, and immune-based therapy are urgently needed to improve patient care. 

Numerous prognostic factors have been suggested to predict survival in ovarian cancer, but tumor stage and residual tumor (<1 cm) after debulking surgery are still considered the most reliable prognostic indicators [8]. Although tumor grading is an important prognostic factor in almost all gynecologic malignancies, it seemed to have a less important value in ovarian cancer. This could be due not to grading itself, but to the lack of a uniform standard that has resulted in little consensus as to whether ovarian tumor grade has any significance in predicting disease outcome. Grading of surface epithelial stromal tumors is still performed haphazardly with several systems and nonsystems used in different institutes and in different research studies. At least five grading systems are in existence; the most commonly used worldwide are the International Federation of Gynecology and Obstetrics (FIGO) system, the World Health Organization (WHO) system, and the Gynecologic Oncology (GOG) system [9, 10]. Silverberg proposed a new grading system similar to that in use in breast carcinoma [11]. It is based on architectural features, cytologic atypia, and mitotic rate. A score is given by adding the parameters: a score of 3–5 is grade 1, a score of 6-7 is grade 2, and a score of 8-9 is grade 3. This grading system was confirmed to be reproducible in subsequent work [12]. Another study from the MD Anderson Cancer Center Group suggested adopting a two-tier system that is based primarily on the assessment of nuclear atypia (uniformity versus pleomorphism) in the worst area of the tumor [13]. The tumor is graded into low grade and high grade. A few years after its introduction, the authors confirmed its reproducibility and urged its use to facilitate the clinical trials and protocols [14, 15].

Even though the origin of epithelial ovarian cancer is still a subject of debate, three anatomical sites, fallopian tube, mesothelial cells covering the peritoneum, and surface ovarian epithelium, have been suggested to be the potential sites of origin for ovarian serous adenocarcinomas [16, 17]. In the last decade, a remarkable advance in understanding the genetic fingerprint of ovarian cancer has been revealed. Two major pathways has been proposed. the first is for low grade serous carcinoma that followed a progression model, which has been defined as a progression from serous borderline tumor (low malignant potential/LMP) to low grade carcinoma, similar to the well-accepted model of adenoma-carcinoma progression of the colon. Low grade serous carcinomas present in younger women and they occur at early stage disease. Although, they have indolent disease course, they are relatively resistant to standard carboplatin and taxol chemotherapy. The second pathway is the* de novo* model consisting of high grade serous carcinomas which present in older women and typically detected as very advanced stage disease. Although they are sensitive to the standard chemotherapy, they are very aggressive disease with a high mortality rate. The genetic pathway in low grade serous carcinoma involves *BARF*, *KRAS*, and *ERBB2* mutations and microsatellite instability (MSI). However, the genetic abnormalities seen in* de novo* high grade serous carcinoma are much more complex as it involves numerous genetic abnormalities. But the most frequent and constant genetic change in high grade serous carcinoma is *p53 *mutations occurring in 50–80% of cases [18]. 

c-MET protooncogene (Gene ID: 4233) is located at 7q31 locus of chromosome 7 [19, 20]. It is a membrane receptor that is essential for embryonic development and wound healing. MET is produced as a single-chain precursor protein and it has an extracellular *α*-submit and transmembrane *β*-subunit which are liked by a bisulfide bond to form a mature receptor. While, the extracellular portion is responsible for binding to hepatocyte growth factor (the only known ligand for MET receptor), the intracellular portion is responsible for signal transduction. The binding of hepatocyte growth factor (HGF)/scatter factor to MET receptor leads to c-Met phosphorylation and therefore to its activation. Once activated, c-Met will interact either directly or indirectly with numerous intracellular substrates such as RAS and Gab1, among others. c-Met engagement activates multiple transduction oncogenic pathways such as the RAS pathway (cell proliferation through MAPK activation), the phosphoinositide 3-kinase (PI3 K) pathway (cell motility through remodeling of adhesion to the extracellular matrix as well as cell survival), the STAT pathway (cell growth, differentiation, and death), and the *β*-catenin pathway (transcriptional regulation of numerous genes that resulted in regulation of cell motility, matrix remodeling, and cell survival and proliferation), as well as the Notch pathway (important in controlling and the regulation of multiple cell differentiation processes during embryonic and adult life) [21, 22]. Furthermore, MET/HGF plays an important role in cancer development which was attributed through numerous mechanisms such as 1-activation of key oncogenic pathways (RAS, PI3 K, STAT, and *β*-catenin), 2-angiogenesis, and 3-scatter (cell dissociation due to metalloprotease production) which leads to invasion and metastasis [23]. However, subsequent studies have shown that the HGF/MET pathway has the capability to interact with other pathways. Example: a cross-talk between transformation growth factor (TGF-*α*)/epidermal growth factor (EGFR) pathway and HGF/MET pathway was recently identified. Phosphorylation of c-Met by TGF-*α*/EGFR (receptor of TGF*α*) was found in several epithelial derived tumor cell lines such as human hepatoma cell lines and epidermoid carcinoma cell line, and subsequently leading to its activation. Most importantly this activation was seen in the absence of HGF [24]. This cross-talk between the TGF*α*/EGFR and HGF/MET could have significant implications for altered growth control in tumorigenesis. However, more studies are needed to evaluate the biological implication in cancer as a result of the cross-talk between these two pathways. 

While MET is normally expressed by epithelial cells, and it is also seen in endothelial cells, neurons, hepatocytes, hematopoietic cells, and melanocytes, HGF is usually expressed in cells of mesenchymal origin, making HGF and MET principal mediators of paracrine epithelial mesenchymal interaction. The MET pathway is abnormally regulated in a wide range of human cancer, including breast, colorectal, lung, pancreatic, lung, and hepatic cancers [25–29]. Aberrant MET signaling seemed to be the results from numerous genetics mechanisms such as germline or somatic gene mutation, c-MET chromosomal rearrangement, c-MET amplification, c-MET transcriptional upregulation, or ligand-dependent autocrine or paracrine changes. MET gene mutation is not a frequent initiating event in most common human cancers, but it was more frequently associated with tumor progression. If we take the case of head and neck squamous cell carcinoma (HNSCC), c-Met mutation was not seen in the primary tumor, but instead it was most likely to be found in lymph node metastasis from HNSCC [30]. In addition, while no mutation was detected in 153 sporadic cancers, somatic c-Met was seen in lymph nodes as well as in lung metastasis from primary tumors such as HNSCC and colorectal carcinomas [31]. These data suggested that c-Met might be an oncogene that is involved in controlling tumor progression and metastasis instead of tumor onset. 

There is compelling evidence on the involvement of HGF/MET pathway in ovarian carcinogenesis. Next, we will discuss the HGF/MET axis alterations in ovarian cancer and its clinical implication, as well as the potential therapeutic use of HGF/MET inhibitors in ovarian cancer.

## 2. HGF/MET Pathway in Ovarian Cancer

### 2.1. MET Expressions in Ovarian Cancer

HGF/MET plays an important role in the development and morphogenesis of urogenital organs. For instance, mice lacking HGF showed impairment in organ development and even died in utero [32]. During embryonal development, HGF was shown to be selectively produced by stromal ovarian cells adjacent to genital ridge, suggesting its involvement in ovarian development and proliferation. MET was expressed in normal ovarian epithelium as well as in benign tumors and it was overexpressed in a subset (30–40%) of epithelial ovarian cancer [33, 34]. Even though this overexpression was seen regardless of the histologic subtypes, it was still most frequently expressed in papillary serous carcinoma and interestingly enough in clear cell carcinoma. The overexpression of MET protein in ovarian carcinoma showed a substantial variation between tumors *versus* normal ovary. Overexpression ranged from 3-fold up to more than 50-fold and this considerable variation was closely associated to HGF induction as seen by Corps and his colleagues [35]. In their experiments, the authors found that SK-OV-3 cell lines expressed high levels of MET protein and marked mitogenic, motogenic, and chemotactic response to added HGF, while CH1 cells expressed lower MET levels and did not respond to HGF. Even more, this response remained unchanged despite a longer incubation time with HGF. The mechanism underlying the difference between the two cell lines is not well understood. However, one thing remained certain, that the involvement of HGF/MET pathway in promoting cell proliferation and dissemination in ovarian cancer was highly associated with the expression of high levels of the HGF receptor. 

MET overexpression was not associated with c-Met mutation, neither with gene amplification. Studying 158 solid tumors including 48 ovarian cancer samples, Lorenzato et al. did not find mutation in the c-Met kinase domain in any of these cases [31]. Based on these results and others in the literature, it was suggested that the alterations of Met axis might not be attributed to a structural abnormality of c-Met, but instead it could be secondary to mutations of other genes, such as RAS (frequently mutated in ovarian cancer), that could influence the HGF/MET pathway [31, 36]. 

### 2.2. HGF/MET Pathway in Ovarian Cancer Transformation

Compelling evidence showed that HGF/MET axis to be responsible of ovarian surface epithelial (OSE) transformation. Since the interaction of stromal-epithelial cell seemed to be vital in the function and growth of normal and tumors of the OSE, a recent study showed that HGF is not only expressed in mesenchymal cells but also in OSE. In this study, HGF-mRNA levels were more elevated in freshly isolated epithelial ovarian cells than in those stromal cells. Also, using immunohistochemistry, HGF was more expressed in epithelial components of ovarian tissue than in stromal components [37]. In addition, OSE cells were not only capable of expressing HGF but to respond to it in an autocrine manner. This unexpected expression of HGF by ovarian epithelial cells led to speculate that these cells have the ability to stimulate their own growth through the unusual autocrine stimulation by HGF. Thus, indicating that while the paracrine interaction of HGF/MET is important in normal ovarian physiology, the autocrine HGF/MET loop seems to be important in ovarian cancer onset and maybe progression. Furthermore, a coexpression of HGF/MET was seen in normal OSE from patients with familial history of ovarian cancer (FH-OSE) compared to those with no familial history (NFH-OSE), suggesting that HGF/MET pathway might play a role in the enhanced susceptibility to ovarian carcinogenesis in women with hereditary ovarian cancer syndromes [38]. 

### 2.3. HGF/MET in Ovarian Progression and Invasion

HGF/SF is the only known ligand for MET receptor. As a growth factor, HGF can stimulate cell proliferation, morphogenesis, cell motility, and invasiveness [39–42]. HGF was highly expressed in ovarian cancer peritoneal ascites and in benign ovarian and cancer cysts fluid [43]. While the benign ascitic fluid contained low levels of HGF and did not simulate migration of ovarian cancer cells, fluids form malignant ovarian cyst and ascitic fluid of women with ovarian cancer were capable of stimulating cancer cell migration and this migration was greatly reduced by the addition of an HGF-neutralized antibody. These results indicated that HGF maybe a major inducer of ovarian cancer cell migration in ovarian tumor [44]. The mechanism of invasion and migration stimulated by HGF was elusive until recent studies discovered a novel mechanism: HGF activates *p*70^*S*6*k*^ which induces matrix metalloproteinase 9 (MMP9) degradation, a matrix protein which is known to be responsible for promoting cellular inavsion and has been found to be associated with poor prognosis in late stage ovarian cancer patients [45–47]. Even more, blocking c-Met using small interfering RNA (siRNA) reduced MET protein and MET mRNA expression as well as inhibited extracellular signal-regulated kinase and PI3 K signaling [48]. In addition, it resulted in a significant reduction of *α*5 and *β*1 integrin protein and urokinase and matrix metalloproteinase 9 (MMP9) activity, and subsequently leading to a reduction of adhesion of cancer cells to human mesothelial cells that cover the peritoneum mainly via reduction of integrin. An* in vivo* study showed an 85% inhibition of the number of tumor nodules, tumor weight, and ascites in mice injected with the c-Met siRNA [49]. Therefore, one might speculate that after tumor debulking, a consolidation therapy might delay the repopulation of the peritoneal cavity by tumor cells deserves serious consideration in future clinical trials. These combined data suggested that by MMP9 inhibition might abolish EGF-HGF induced cellular invasiveness and, therefore, it could be a potential target therapy for ovarian cancer. 

HGF/MET pathway has been implicated in tumor angiogenesis through the effect of HGF on proangiogenic factors such as VEGF, interleukin 8, and thrombospondin 1. However, in ovarian cancer, it seemed that HGF/MET did not regulate angiogenesis [49]. This might be due to the difference in the mode of dissemination that exists between ovarian cancer/peritoneal spreading and other solid tumors/lymphovascular [17]. 

### 2.4. The Effect of the Cross-Talk between HGF/MET Pathway and Other Pathways

Cross-talk between cell surface receptors had gained a major interest in carcinogenesis. A cross-talk between the *RON *gene (a member of the receptor tyrosine kinase gene family that includes c-Met oncogene) and c-Met has been identified in ovarian cancer.In a study by Maggiora et al., *RON* and c-Met oncogenes were coexpressed in 42% of ovarian cancer cases and it has been suggested that this coexpression might cooperate in promoting ovarian cancer progression [50].

Another cross-talk was seen between HGF/MET pathway and epidermal growth factor (EGF) pathway. HGF and the epidermal growth factor (EGF) were found to use unique as well as overlapping signaling cascades leading to invasiveness phenotype. The mechanism of invasive activity of HGF was revealed to be through the activation of PI3 K/Akt and extracellular signal-regulated kinase (ERK) 1/2. In addition, it seemed that EGF and HGF cooperate to promote invasiveness in ovarian cancer cell lines through increase secretion of MMP9 and the inhibition of MMP9 abolished EGF and HGF induced cellular invasion [51]. Once again, these results data indicate that HGF/MET overexpression has a significant role in promoting cell migration and matrix degradation and therefore ovarian cancer progression.

### 2.5. HGF/MET Effect on Standard Chemotherapy in Ovarian Cancer

Paclitaxel and cisplatin are the standard chemotherapy used to treat patients with ovarian cancer. Therefore, it would make sense to evaluate the effect of HGF on ovarian cancer cell response to paclitaxel and cisplatin. The primary intracellular targets of paclitaxel and cisplatin are distinct but both prompt apoptosis and activate the MAPK pathway. Unlike other cancers where HGF is known to protect tissues from cytotoxic effects of chemotherapeutic agents, Rasola et al. in a 2004 study showed that HGF sensitizes ovarian cancer cells to low dose chemotherapy [52]. This unexpected result led the authors to suggest that HGF may be used to improve the response to chemotherapy in human ovarian carcinoma over-expressing c-Met. The mechanism explaining this effect was later attributed to the activation of *p38* MAPK signaling pathway [53]. In a series of studies, it has been shown that HGF triggers survival signaling pathways in ovarian cancer cells, but this survival effect is overwhelmed by the dominant role of the apoptotic effect of *p38* MAPK on the treated cells. These data indicated that *p38* MAPK pathway might be a candidate target to the sensitization of ovarian cancer cells to low dose chemotherapeutic drugs. However, in 2010, Tang et al. showed that using three-dimensional ovarian cell cultures, MET overexpression enhanced the survival of cancer cells and increased resistance to cisplatin and paclitaxel chemotherapeutic agents [54]. Also inhibition of c-Met by siRNA blocked the anoikis and apoptosis resistance and restored chemosensitivity in three-dimensional but not in two-dimensional cell cultures. These effects were found to be dependent on both PI3k/Akt and the ERK1/2 signaling pathways. These contradictory findings betwen the above data might be due to the conditions of cell cultures; such as monolayer/bidimensional versus three-dimensional cell cultures. While *in vivo* findings are very encouraging, they still have not been translated into successful therapies. Still, HGF/MET involvement in responsiveness to the standard chemotherapeutic agents has yet to be settled and it is up to a future study to confirm either of these two findings.

## 3. Clinical Implication

In ovarian cancer, overexpression of MET was associated with poor prognosis where tumors with overexpression of MET protein had lower survival rate in comparison to those with low MET expression (17 months versus 32 months) [49]. In one study MET overexpression was seen in premenopausal patients suggesting that its overexpression could be under hormonal control, but this theory has yet to be determined [34].

Using single-nucleotide polymorphism (SNP) on samples from Mayo clinics and the Tumor Cancer Genomic Atlas (TCGA), Goode et al. examined whether mortality was associated with inherited variation in 170b candidate genes/regions [55]. The authors found evidence of an association between variants in *HGF* and increased mortality in samples from both Mayo clinics and the TCGA. However, when they evaluated HGF, MET, and phospho-MET protein expression using immunohistochemistry in a tissue microarray (TMA), the authors did not observe a clear relationship between SNP genotype, expression of proteins, and outcomes. Yet this study only confirms once again the complexity and the challenges of molecular epidemiologic investigations. 

## 4. HGF/MET Therapeutic Targets

Because of its ubiquitous role in cancer, the MET axis makes it an attractive target for cancer therapy. The majorities of MET inhibitors are still in phase I and phase II trials, but a few compounds are in phase III trials in lung and medullary thyroid cancer [56–63]. Several MET pathway inhibitors are currently being studied and these agents focus on the various steps that lead to MET activation including (1) antibodies that compete and block the binding of HGF to MET and therefore blocking downstream activation of the pathway that is, rilotumumab and ficlatuzumab; (2) monoclonal antibodies that block the activation of MET receptor. By binding to the receptor, these antibodies resulted to its degradation and subsequently to its inactivation (i.e., onartuzumab); (3) Selective MET kinase inhibitors that inhibit MET receptor activation such as tivantinib (ARQ 197) and PF04217903 and nonselective MET kinase inhibitors such as crizotinib (PF02341066), cabozantinib (XL 184), and foretinib. 

Few compounds have been explored in ovarian cancer such as (1) NK4: NK4 is a HGF/SF fragment that competes with HGF/SF for binding to c-Met receptor in ovarian cancer cell lines. Studies showed that NK4 could inhibit ovarian cancer cell migration, tumor growth, and peritoneal dissemination *in vivo*, indicating that NK4 could be an attractive target for therapy in inhibiting HGF/MET pathway [64]. (2) MMP9 inhibitors as already discussed previously could be a potential therapeutic candidate. (3) An orally available small molecule inhibitor of c-MET, PF-2341066 was evaluated in mice model by Zillhart and his colleagues [65]. Results showed that after being injected intraperitoneally with the ovarian cancer cell line (SKOV3ip1), treatment of mice with PF-2341066 resulted in the reduction of tumor burden, tumor weight, and the number of metastasis by 55%. In addition, PF-2341066 inhibited proliferation and adhesion to various extracellular matrix as well as reduction of the activity of matrix metalloproteinase. (4) In another study by the same group of researchers using foretinib (GSK1363089), found that foretinib was able to prevent the progression of primary tumors to invasive cancer in genetic mouse model by completely blocking the invasion of tumor cells through the basement membrane. Treating the xenograft mouse model using human ovarian cancer cell lines with oral foretinib, there was a reduction of tumor burden (86%) and metastasis (67% inhibition). This effect involved numerous mechanisms such as inhibition of c-Met activation and downstream signaling, reduction of cancer cell adhesion, blocking migration and invasion, reducing proliferation, and induction of anoikis [66]. 

Knowing that ovarian cancer is a disease with very limited therapeutic options, the above preliminary data suggest that MET inhibitors compounds seem to be very promising therapeutic candidates and they deserve serious consideration for further clinical development in treating patients with ovarian cancer. Hopefully, MET inhibitors by themselves or in combinations with the standard chemotherapy would be seen in clinical trials in the future.

## 5. Conclusion

 HGF/MET pathway plays a major role in ovarian cancer onset and progression including invasiveness and metastasis. The inhibition of HGF/MET pathway would block the downstream effect of this pathway, including RAS pathway, PI3 K pathway, STAT pathway, *β*-catenin pathway, and Notch pathway. This is an exciting era in oncology and hopefully more studies on monoclonal inhibitor antibodies would be conducted in patients with ovarian cancer. Only the results in the coming years will tell on the efficacy of MET inhibitors in solid tumors, namely, ovarian cancer.
